# Regional patterns of incidental pelvic nodal irradiation during bladder-only radiotherapy: A paired dosimetric analysis of VMAT and IMRT

**DOI:** 10.1016/j.ctro.2026.101273

**Published:** 2026-08-29

**Authors:** Birhan Demirhan, Aysenur Elmali, Recep Bozca, Yemliha Dolek, Ozan Cem Guler, Cem Onal

**Affiliations:** a Division of Radiation Oncology, Iskenderun Gelisim Hospital, Hatay, Turkiye; b Department of Radiation Oncology, Baskent University Faculty of Medicine, Ankara, Turkiye; c Department of Radiation Oncology, Baskent University Faculty of Medicine Adana Dr. Turgut Noyan Research and Treatment Center, Adana, Turkiye

**Keywords:** Bladder cancer, radiotherapy, pelvic lymph nodes, incidental nodal irradiation, VMAT, IMRT

## Abstract

**Purpose:**

Incidental pelvic nodal irradiation is an unavoidable consequence of bladder-only radiotherapy (RT) and may contribute to the irradiation of occult microscopic disease. We characterized incidental pelvic nodal dose and compared regional dose distributions between volumetric modulated arc therapy (VMAT) and step-and-shoot intensity-modulated RT (IMRT).

**Materials and Methods:**

Planning computed tomography datasets from 30 patients receiving bladder-only RT were used to generate paired VMAT and step-and-shoot IMRT plans. Pelvic nodal regions, including the common iliac, external iliac, internal iliac, obturator, and presacral basins, were contoured according to consensus guidelines. Composite and region-specific dose–volume histogram parameters were compared between techniques using paired analyses. Treatment delivery efficiency was evaluated by monitor units (MUs).

**Results:**

Both techniques achieved equivalent target coverage and comparable incidental pelvic nodal dose distributions. A consistent regional hierarchy of incidental nodal irradiation was observed, with the obturator, external iliac, internal iliac, and common iliac regions receiving approximately 75%, 45%, 25%, and < 5% of the prescribed dose, respectively. These patterns remained remarkably consistent regardless of delivery technique. Although small statistically significant differences were identified for selected regional dose–volume parameters, the magnitude of these differences was generally less than 1 Gy and was considered unlikely to be clinically meaningful. VMAT required significantly fewer MUs than step-and-shoot IMRT (median 914 vs. 1123, *p* < 0.001).

**Conclusions:**

Bladder-only RT produces a reproducible regional pattern of incidental pelvic nodal irradiation that is largely independent of delivery technique. The observed consistency across techniques suggests that incidental nodal exposure may be influenced more by target geometry than by the delivery technique.

## Highlights

• Bladder-only radiotherapy produces predictable incidental pelvic nodal irradiation.
• Obturator nodes receive the highest incidental radiation exposure.
• Regional dose hierarchy is independent of IMRT delivery technique.
• Target geometry determines incidental nodal dose more than technique.
• VMAT improves delivery efficiency without altering nodal dose.

## 1 Introduction

Bladder-preserving trimodality therapy is an established treatment option for selected patients with muscle-invasive bladder cancer (MIBC), providing oncologic outcomes comparable to radical cystectomy while preserving bladder function [1], [2]. However, the role of elective pelvic nodal irradiation (ENI) during bladder-preservation radiotherapy (RT) remains controversial. Although ENI has been advocated because of the risk of occult microscopic nodal disease and predictable patterns of pelvic lymphatic spread [3], its routine use has been limited by the lack of randomized evidence demonstrating a survival benefit and concerns regarding treatment-related toxicity [4], [5], [6].

Pelvic nodal involvement is common in muscle-invasive bladder cancer and increases with advancing tumor stage. In a contemporary pathological mapping study of 1555 patients undergoing radical cystectomy and pelvic lymph-node dissection, nodal metastases were identified in 19.1% of patients overall, increasing from 11.0% in T2 disease to 29.8% in T3 and 50.0% in T4 disease [7]. Region-specific pathological analyses further demonstrated that metastatic involvement occurs predominantly in the obturator, external iliac, and internal iliac nodal basins [8], [9]. This anatomical distribution raises the possibility that incidental irradiation during bladder-only RT may preferentially expose nodal regions at greatest risk of occult microscopic disease.

Modern bladder RT is predominantly delivered using intensity-modulated radiotherapy (IMRT), most commonly as volumetric modulated arc therapy (VMAT) or fixed-field IMRT [10], [11]. Both techniques provide highly conformal target coverage while reducing normal tissue dose, with VMAT additionally improving treatment efficiency through shorter delivery times and fewer monitor units (MUs) [12], [13]. Consequently, previous dosimetric studies have focused primarily on target coverage, organ-at-risk (OAR) sparing, and treatment efficiency, whereas incidental irradiation of adjacent pelvic lymphatic regions has received relatively little attention [14], [15], [16], [17], [18].

Because of the close anatomical relationship between the bladder and pelvic lymphatic drainage pathways, incidental pelvic nodal irradiation is an unavoidable consequence of bladder-only RT. Such unintended irradiation may be clinically relevant, particularly in patients with occult microscopic nodal disease, and may partly explain the favorable pelvic control reported after bladder-preservation despite omission of ENI [3], [4], [5], [6], [19], [20]. However, the magnitude and regional distribution of incidental nodal irradiation remain poorly defined. Previous studies have largely evaluated pelvic lymph nodes as a composite structure, providing limited insight into dose heterogeneity among individual nodal basins or the influence of contemporary IMRT delivery techniques on regional dose patterns [19], [20].

To our knowledge, this is the first study comparing regional incidental nodal irradiation across individual pelvic nodal basins using paired VMAT and step-and-shoot IMRT planning. We therefore performed a paired dosimetric analysis to quantify incidental nodal doses in both composite pelvic nodal volumes and individual nodal basins and to evaluate whether regional dose patterns differ according to delivery technique. We hypothesized that bladder-only RT produces a reproducible regional hierarchy of incidental nodal irradiation, with preferential dose deposition in anatomically adjacent nodal basins irrespective of IMRT delivery technique.

## 2 Materials and methods

### 2.1 Study design and patient selection

This retrospective dosimetric study was designed as a paired within-patient comparison of RT delivery techniques. Planning computed tomography (CT) datasets from 30 consecutive patients with bladder cancer previously treated with definitive RT at our institution were included. For each patient, two separate treatment plans, VMAT and step-and-shoot IMRT, were generated on the same CT dataset, thereby enabling direct dosimetric comparison under identical anatomical and planning conditions. The study was conducted exclusively for dosimetric evaluation and did not include clinical outcome analyses.

The study was conducted in accordance with applicable laws, regulatory frameworks, and institutional guidelines. Under these regulations and guidelines, ethics committee approval and informed consent were not required because the study used fully anonymized planning CT datasets and involved no additional intervention or collection of identifiable patient information.

### 2.2 Simulation and imaging protocol

All patients underwent CT simulation in the supine position using standardized immobilization. Images were acquired with a 2.5-mm slice thickness and imported into the treatment planning system. A standardized bladder-filling protocol consisting of oral hydration with 500 mL of water approximately 30 min before CT simulation was used to improve bladder reproducibility. The same bladder-filling protocol was followed before each treatment fraction. Patients were instructed to empty their bowel before imaging, no additional bowel preparation was performed.

### 2.3 Target volume and nodal delineation

Target volumes were defined according to institutional standards. The clinical target volume (CTV) included the entire bladder. The planning target volume (PTV) was generated using a uniform 1.5-cm expansion, consistent with the institutional non-adaptive bladder RT protocol used during the study period. This margin was selected to account for interfractional variations in bladder volume, shape, and position. No adaptive replanning strategy was employed. Daily cone-beam CT was performed before each treatment fraction for image-guided verification and patient-position correction.

Pelvic lymph node regions were prospectively contoured to evaluate incidental nodal irradiation. To ensure consistency, all nodal delineations were performed by a single investigator (O.C.G.) according to published consensus guidelines [21], [22]. Nodal regions were generated using a 5-mm expansion around the corresponding pelvic vessels while excluding adjacent non-nodal structures. Surrounding adipose tissue at risk for microscopic disease extension was included where appropriate.

**Fig. 1:**
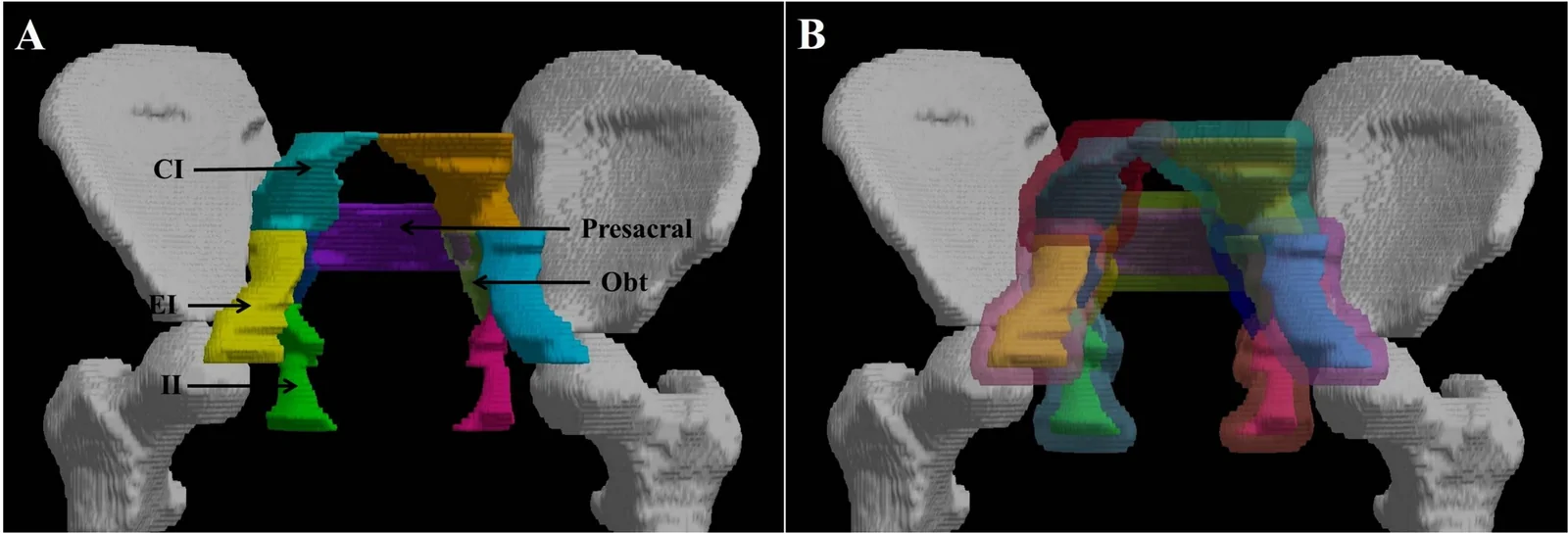
Three-dimensional figures illustrating the delineation of individual pelvic lymph node subregions used for dosimetric analysis. (A) Individual nodal subregions contoured according to consensus guidelines, including common iliac (CI), external iliac (EI), internal iliac (II), obturator (Ob), and presacral regions. (B) Corresponding planning target volumes (PTVs) of nodal subregions.

The pelvic nodal CTV comprised the common iliac, external iliac, internal iliac, obturator, and presacral nodal regions (Fig. 1). Nodal structures were analyzed both as a composite volume and as individual nodal basins, with right- and left-sided regions evaluated separately where applicable.

### 2.4 Treatment planning

All plans were generated using the Monaco treatment planning system (version 5.51.10; Elekta, Stockholm, Sweden) with a Monte Carlo dose calculation algorithm and were designed for delivery on an Elekta Versa HD linear accelerator using 6-MV photon beams. The prescribed dose was 66 Gy in 33 fractions.

VMAT and step-and-shoot IMRT plans were optimized using identical planning objectives and OAR constraints to achieve comparable target coverage and dose homogeneity. VMAT plans consisted of two coplanar arcs, whereas step-and-shoot IMRT plans used 11 fixed gantry fields with inverse optimization.

### 2.5 Dose–volume histogram analysis

Dose–volume histograms (DVHs) were generated for both treatment plans. Target dosimetry included D2, D98, V95, V107, conformity index (CI), and homogeneity index (HI).

Plan quality was further evaluated using the CI and HI, which were compared between the two techniques. The CI was calculated according to the following formula:

$$\mathrm{CI}=\frac{{\mathrm{VT}}_{\mathrm{ref}}}{\mathrm{TV}}\times \frac{{\mathrm{VT}}_{\mathrm{ref}}}{V_{\mathrm{ref}}},$$

where ${\mathrm{VT}}_{\mathrm{ref}}$ is the target volume covered by the isodose, TV is the total target volume, and $V_{\mathrm{ref}}$ is the volume covered by 95% of the isodose [23]. The CI ranges from 0 to 1, with a value closer to 1 indicating better dose conformity to the PTV.

The HI was calculated as follows:

$$\mathrm{HI}=\frac{D_{2-}D_{98}}{D_{50}}\;,$$

where $D_{2}$ and $D_{98}$ were used as surrogates for maximum and minimum doses [24]. A higher HI value indicates greater non-uniformity in dose distribution.

For the composite pelvic nodal volume and each individual nodal basin, evaluated parameters included Dmean, Dmax, D100, D66, D33, V40, and V50. All dose calculations were performed using the Monte Carlo algorithm with a statistical uncertainty ≤1%. Treatment delivery efficiency was assessed by total MUs.

### 2.6 Statistical analysis

All statistical analyses were performed using SPSS version 25.0 (IBM Corp., Armonk, NY, USA). No formal a priori sample-size or statistical power calculation was performed. This exploratory analysis included 30 consecutive eligible patients, with the paired within-patient design allowing each patient to serve as their own control. Because several dosimetric parameters demonstrated non-normal distribution, comparisons between VMAT and step-and-shoot IMRT were performed using the Wilcoxon signed-rank test. Continuous variables are reported as median and interquartile range (IQR) or mean ± standard deviation, as appropriate. To reduce the risk of type I error related to multiple regional comparisons, primary emphasis was placed on composite nodal endpoints, whereas region-specific analyses were considered exploratory. All statistical tests were two-sided, and a *p*-value <0.05 was considered statistically significant.

## 3 Results

### 3.1 Target coverage and plan quality

Both VMAT and step-and-shoot IMRT achieved all institutional planning objectives, providing equivalent target coverage and overall plan quality. No significant differences were observed in PTV dose–volume parameters, including V95, V107, D2, D98, mean target dose, conformity index, or homogeneity index (Table 1). These findings confirmed that subsequent comparisons of incidental nodal irradiation were performed between dosimetrically equivalent treatment plans.

**Table 1:** Bladder target dose–volume parameters for VMAT and step-and-shoot IMRT plans.

Parameters	VMAT plan (mean ± SD)	IMRT plan (mean ± SD)	*p*
V_107_ (%)	0.23 ± 0.50	0.26 ± 0.07	0.56
V_95_ (%)	98.65 ± 0.97	98.39 ± 0.89	0.79
D_2_ (Gy)	71.63 ± 0.36	71.38 ± 0.32	0.43
D_98_ (Gy)	62.14 ± 0.89	62.22 ± 0.82	0.52
D_mean_ (Gy)	67.80 ± 0.25	67.84 ± 0.42	0.72
CI	0.79 ± 0.04	0.74 ± 0.03	0.11
HI	0.14 ± 0.02	0.13 ± 0.02	0.46

Abbreviations: VMAT = volumetric modulated arc therapy; IMRT = intensity-modulated radiotherapy; SD = standard deviation; V = volume; D = dose; Gy = gray; CI = conformity index; HI = homogeneity index.

**Fig. 2:**
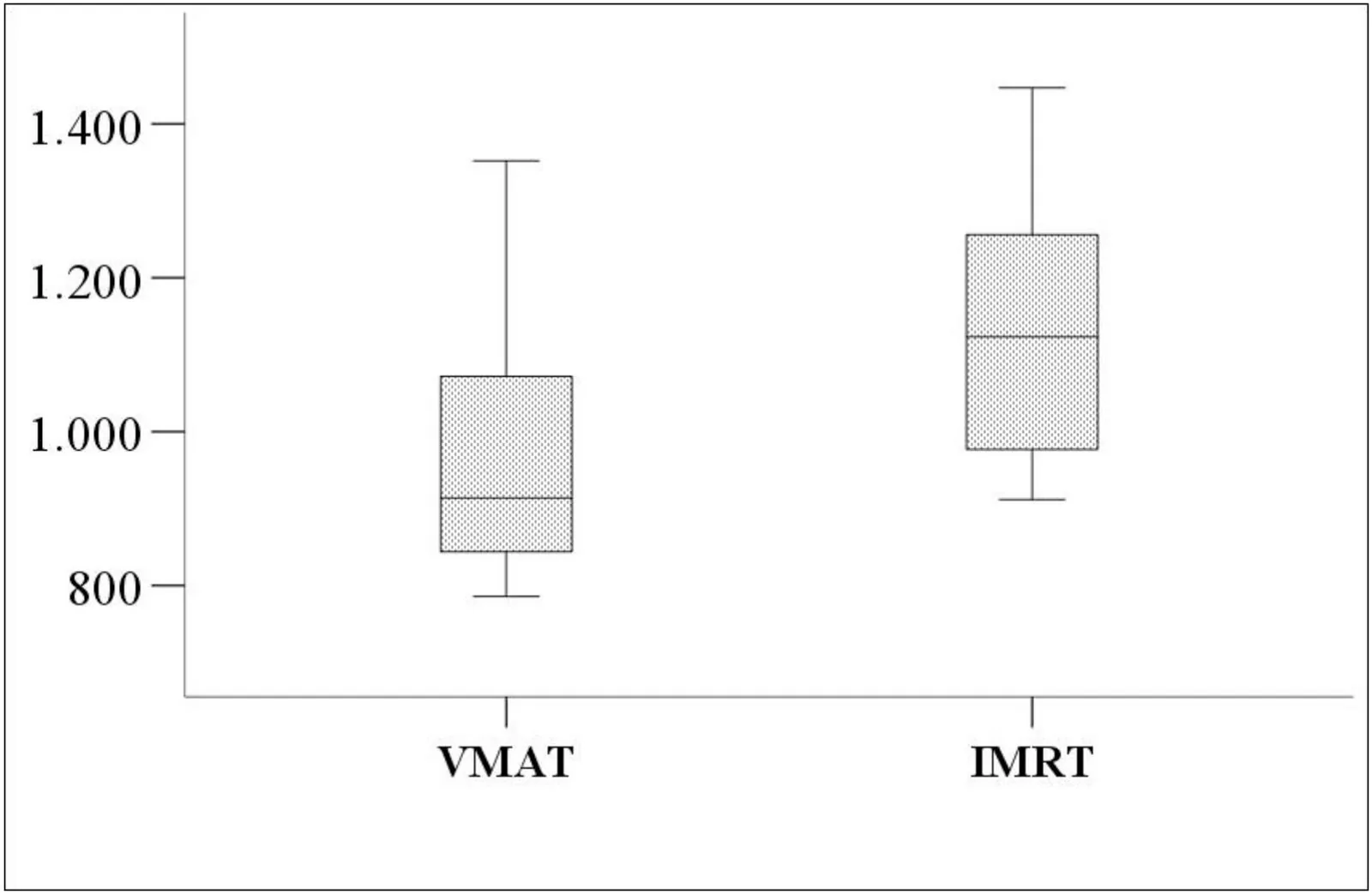
Box-and-whisker plot comparing total monitor units (MUs) required for VMAT and step-and-shoot IMRT plans.

VMAT significantly improved treatment delivery efficiency, requiring fewer MUs than step-and-shoot IMRT (median 914 [IQR, 844–1073] vs. 1123 [IQR, 977–1258]; *p* < 0.001). Step-and-shoot IMRT required more MUs in 29 of 30 patients (Fig. 2).

### 3.2 Regional pattern of incidental pelvic nodal irradiation

**Fig. 3:**
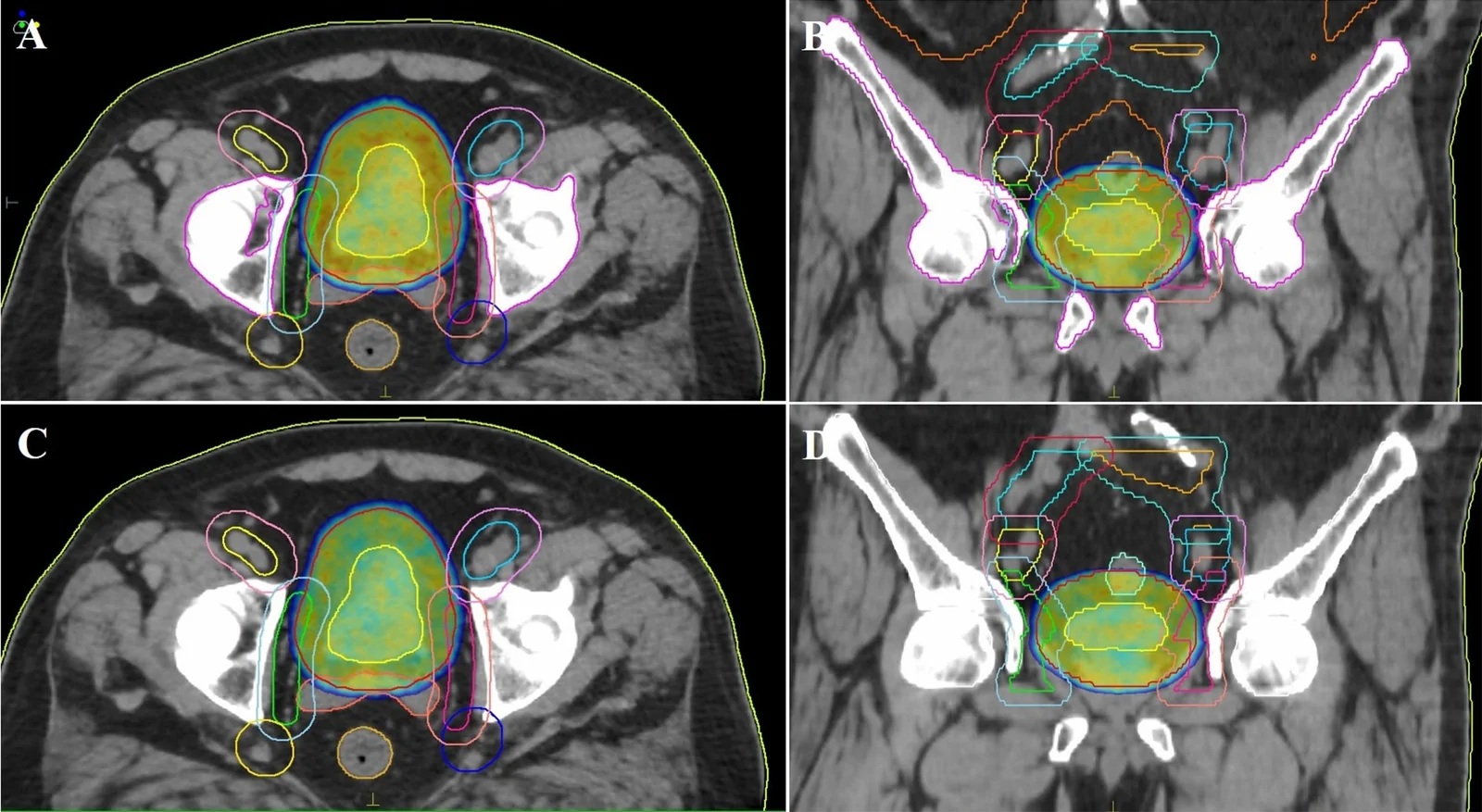
Representative axial and coronal computed tomography images showing 90% dose distributions of prescribed dose and contoured bladder target and pelvic nodal subregions for VMAT (A, B) and step-and-shoot IMRT (C, D).

**Fig. 4:**
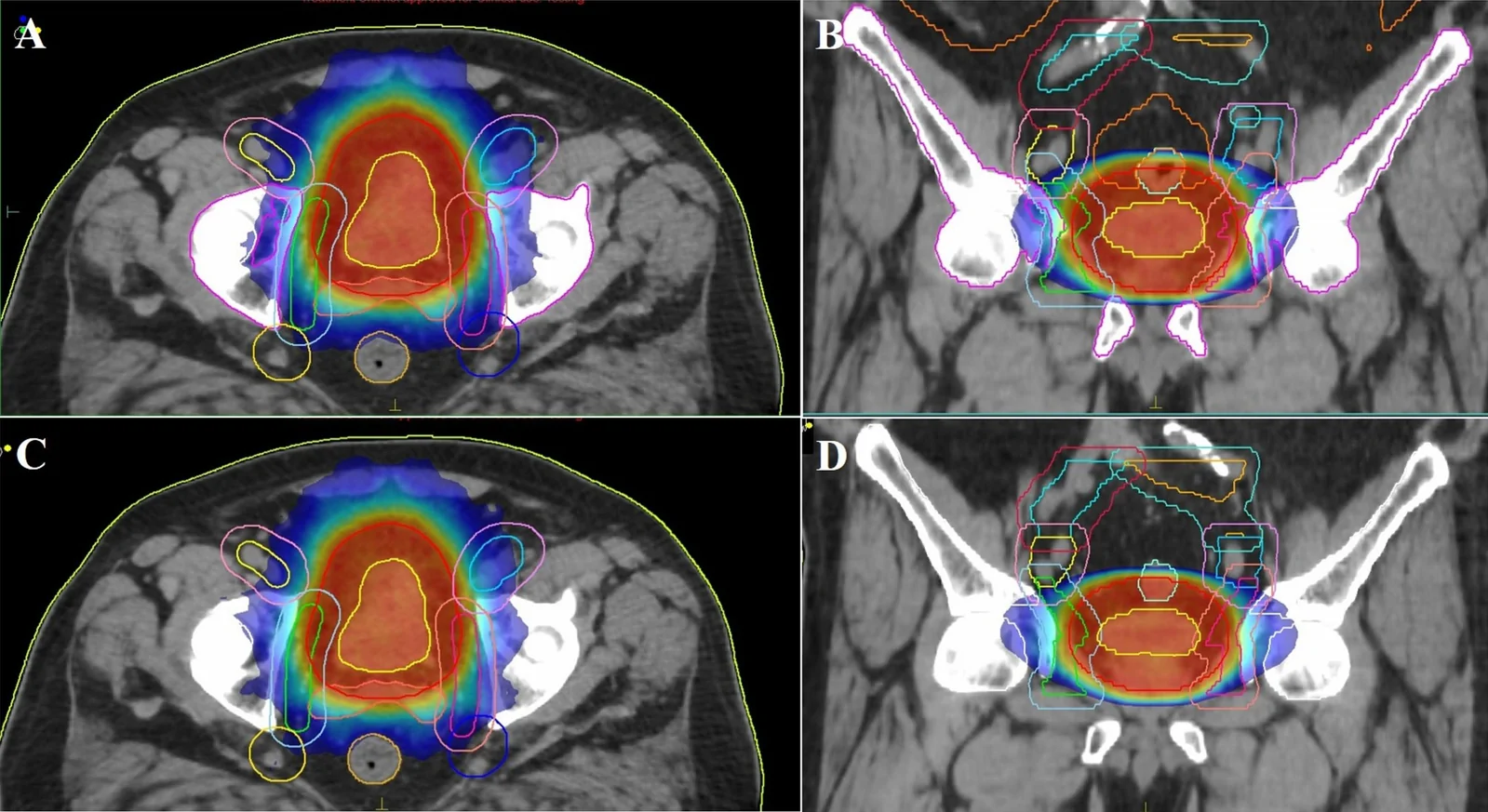
Representative axial and coronal computed tomography images showing 50% dose distributions of prescribed dose and contoured bladder target and pelvic nodal subregions for VMAT (A, B) and step-and-shoot IMRT (C, D).

Bladder-only RT produced a consistent regional hierarchy of incidental pelvic nodal irradiation irrespective of delivery technique. Across both VMAT and step-and-shoot IMRT plans, the obturator nodal regions consistently received the highest incidental doses, followed by the external iliac and internal iliac basins, whereas common iliac nodes received minimal irradiation. Differences in composite pelvic nodal dosimetry between the treatment techniques were unlikely to be clinically meaningful. Although isolated statistically significant differences were observed for selected regional dose–volume parameters, these differences were small (generally <1 Gy), inconsistent across nodal regions, and unlikely to be clinically meaningful (Fig. 3, Fig. 4).

### 3.3 Effect of delivery technique on incidental nodal dose

Despite differences in beam delivery, VMAT and step-and-shoot IMRT produced highly comparable incidental pelvic nodal dose distributions. Differences in composite nodal dose–volume parameters between the techniques were considered unlikely to be clinically meaningful. Although isolated statistically significant differences were observed for selected regional dose–volume metrics, these differences were small (generally <1 Gy), inconsistent across nodal basins and laterality, and were not accompanied by corresponding changes in related dose–volume parameters. Consequently, no systematic dosimetric advantage of either delivery technique was identified (Table 2).

**Table 2:** Comparison of incidental dose–volume histogram (DVH) parameters for individual pelvic lymph node subregions between VMAT and step-and-shoot IMRT. Right and left nodal basins (common iliac, external iliac, internal iliac, and obturator) are reported separately.

Parameter	VMAT (Median, IQR)	IMRT (Median, IQR)	*p*	VMAT (Median, IQR)	IMRT (Median, IQR)	*p*
	***Right common iliac***			***Left common iliac***		
Dmean (Gy)	1.89 (1.29–6.06)	1.69 (1.28–5.93)	0.03	1.99 (1.39–5.88)	1.97 (1.29–5.38)	0.04
Dmax (Gy)	4.23 (2.95–52.73)	4.10 (3.00–48.48)	0.06	4.72 (3.20–52.13)	4.55 (3.00–49.98)	<0.001
V40Gy (%)	0 (0–0.45)	0 (0–1.75)	0.78	0 (0–0.30)	0 (0–0.07)	<0.001
V50Gy (%)	0 (0–0.01)	0 (0–0.01)	1.00	0 (0)	0 (0)	0.78
	***Right external iliac***			***Left external iliac***		
Dmean (Gy)	30.00 (21.50–45.92)	30.78 (22.17–44.62)	0.04	30.00 (21.50–45.93)	29.05 (22.58–44.55)	0.004
Dmax (Gy)	69.90 (67.72–70.62)	69.80 (68.05–70.70)	0.53	69.90 (67.72–70.62)	69.85 (67.67–70.35)	0.95
V40Gy (%)	36.90 (17.87–62.00)	37.23 (17.92–60.95)	0.19	13.80 (2.19–48.87)	12.85 (2.05–48.48)	0.01
V50Gy (%)	21.85 (7.82–43.00)	21.00 (8.30–44.97)	0.13	2.95 (0–29.92)	3.10 (0.08–29.78)	0.43
	***Right internal iliac***			***Left internal iliac***		
Dmean (Gy)	18.55 (12.25–36.52)	18.85 (12.58–36.35)	0.08	17.00 (13.87–33.10)	16.7 (13.37–32.70)	0.12
Dmax (Gy)	63.45 (51.94–69.90)	64.10 (52.75–70.03)	0.51	54.85 (49.10–69.60)	57.60 (50.35–69.37)	0.29
V40Gy (%)	13.80 (2.19–48.87)	12.85 (2.05–48.48)	0.01	6.05 (2.45–34.12)	7.80 (2.48–35.05)	0.02
V50Gy (%)	2.95 (0–29.92)	3.10 (0.08–29.78)	0.43	0.25 (0–28.82)	1.17 (0–17.47)	0.49
	***Right obturator***			***Left obturator***		
Dmean (Gy)	51.05 (44.45–58.87)	51.10 (45.07–55.97)	0.73	51.10 (44.80–53.21)	50.60 (45.37–53.52)	0.92
Dmax (Gy)	70.50 (70.40–71.02)	70.40 (70.10–70.72)	0.11	70.45 (70.18–70.85)	70.45 (70.10–71.17)	0.49
V40Gy (%)	74.50 (61.17–86.12)	73.50 (59.87–84.32)	<0.001	73.05 (57.55–81.17)	70.25 (56.77–81.75)	0.42
V50Gy (%)	53.60 (43.97–69.82)	57.10 (44.75–69.95)	0.22	53.40 (39.90–60.12)	52.55 (40.00–61.12)	0.12

Abbreviations: VMAT = volumetric modulated arc therapy; IMRT = intensity-modulated radiotherapy; IQR = interquartile range; Dmean = mean dose; Dmax = maximum dose; V40 Gy = percentage of volume receiving ≥ 40 Gy; V50 Gy = percentage of volume receiving ≥ 50 Gy; Gy = gray.

## 4 Discussion

In this paired dosimetric study, we demonstrated that bladder-only RT produces a reproducible regional pattern of incidental pelvic nodal irradiation. The highest incidental doses were consistently delivered to the obturator nodal regions, followed by the external and internal iliac nodal basins, whereas common iliac nodes received minimal irradiation. Importantly, this regional hierarchy remained remarkably consistent regardless of whether treatment was delivered using VMAT or step-and-shoot IMRT, suggesting that incidental nodal exposure may be influenced more by target geometry than by the beam delivery strategy. Although VMAT significantly improved treatment delivery efficiency by reducing MU requirements, the observed differences in incidental nodal dose distribution were unlikely to be clinically meaningful.

The role of ENI in bladder-preservation therapy remains controversial. Although pelvic lymph nodes represent a common site of microscopic disease dissemination, routine ENI has not been universally adopted because randomized studies have failed to demonstrate a clear survival advantage, while concerns regarding treatment-related toxicity persist [4], [25], [26]. Consequently, understanding the extent of incidental nodal irradiation during bladder-only RT is clinically relevant. Our findings demonstrate that bladder-only RT does not equate to complete avoidance of pelvic nodal irradiation. Instead, anatomically adjacent nodal basins, particularly the obturator and external iliac regions, receive substantial incidental radiation exposure, whereas more cranial nodal stations receive considerably lower doses. This observation suggests one potential biological explanation for the favorable pelvic control reported after bladder-preservation strategies despite omission of elective nodal irradiation.

Previous studies investigating incidental pelvic nodal irradiation during bladder RT are limited. Lewis et al. [19] first demonstrated that bladder-only RT delivers clinically relevant incidental doses to pelvic lymph nodes, raising the possibility that unintended nodal irradiation may contribute to regional disease control. More recently, Ozyigit et al. [20] confirmed these observations using contemporary IMRT planning and reported substantial incidental irradiation of selected pelvic nodal regions during bladder-only treatment. In contrast to previous studies that primarily evaluated composite nodal volumes, our study compared regional incidental nodal irradiation across individual pelvic nodal basins using paired VMAT and step-and-shoot IMRT plans generated under identical anatomical and planning conditions. Our findings extend the existing evidence by demonstrating a reproducible regional hierarchy of incidental pelvic nodal irradiation that is largely independent of the delivery technique.

The observed dosimetric hierarchy also demonstrates notable anatomical concordance with established patterns of pelvic nodal spread in bladder cancer. In a pathological series of 162 node-positive patients, 38% of nodal metastases were located in the obturator region, 33% in the external iliac region, and 29% in the internal iliac region [7]. More recent sentinel-node mapping likewise identified the obturator fossa as the most frequent site of nodal metastasis [9]. Thus, the nodal basins receiving the greatest incidental doses in our study closely parallel to regions most commonly involved by metastatic disease. Although this dosimetric analysis cannot establish that incidental irradiation provides regional control or improves clinical outcomes, this anatomical concordance supports the potential biological and clinical relevance of the observed dose distribution.

The observed differences in incidental nodal irradiation between VMAT and step-and-shoot IMRT were unlikely to be clinically meaningful. Although these techniques differ in beam modulation, gantry motion, and dose delivery mechanics, both were optimized using identical target volumes and planning objectives. The consistency of regional dose patterns between the two techniques suggests that dose deposition in adjacent pelvic nodal structures may be influenced more by the geometry of the planning target volume and surrounding anatomy than by the specific beam delivery approach. The small statistical differences observed in selected dose–volume parameters were generally less than 1 Gy, were inconsistent across nodal regions, and were not accompanied by corresponding changes in related dose metrics, suggesting that they are unlikely to be clinically meaningful.

From a clinical perspective, our findings have several implications. First, they indicate that incidental pelvic nodal irradiation should be considered when interpreting historical and contemporary bladder-preservation studies that employed bladder-only RT. Second, they suggest that the biological distinction between bladder-only RT and formal ENI may be smaller than commonly assumed for nodal regions immediately adjacent to the bladder, particularly the obturator and external iliac basins. Finally, these data may help inform the design and interpretation of future clinical trials evaluating the role of ENI by providing a clearer understanding of the baseline nodal dose already delivered during contemporary bladder-only RT. Whether these incidental doses translate into improved regional control or influence oncologic outcomes remains uncertain and warrants prospective investigation.

Consistent with previous dosimetric comparisons across multiple disease sites, VMAT significantly improved treatment delivery efficiency by reducing MU requirements while maintaining equivalent target coverage and dose conformity [14], [16], [17], [18], [27], [28], [29], [30], [31]. Shorter treatment delivery may reduce the impact of intrafraction organ motion and improve treatment reproducibility, particularly in bladder RT where bladder filling variability may influence target position [27], [29]. However, our findings indicate that these technical advantages do not substantially influence incidental pelvic nodal dose distribution.

Several limitations should be acknowledged. First, this was a retrospective dosimetric study without clinical outcome data; therefore, the relationship between incidental nodal irradiation and regional tumor control cannot be determined. In addition, no formal a priori sample-size calculation was performed, and the relatively small cohort may have limited the ability to detect small differences between the planning techniques. Second, all treatment plans were generated using a single institutional planning protocol, which may limit generalizability to other contouring strategies or optimization approaches. Furthermore, the uniform 1.5-cm PTV margin used in this non-adaptive protocol is relatively large by contemporary standards and may have increased incidental nodal exposure, potentially limiting the generalizability of our findings to adaptive strategies or protocols using smaller margins. Third, multiple regional and dose–volume comparisons were performed, increasing the possibility of type I error despite the predefined emphasis on composite nodal endpoints. Finally, although the observed incidental doses may be biologically relevant, the threshold dose required to eradicate occult microscopic nodal disease in bladder cancer remains unknown.

Despite these limitations, the present study has several notable strengths. The paired within-patient design eliminated interpatient anatomical variability, allowing direct comparison of treatment techniques under identical planning conditions. Furthermore, comprehensive contouring of individual pelvic nodal basins according to consensus guidelines enabled a detailed characterization of regional incidental nodal irradiation beyond the composite analyses reported previously. Together, these features provide a robust assessment of incidental pelvic nodal dose during contemporary bladder-only RT.

## 5 Conclusions

Bladder-only RT consistently delivers substantial incidental irradiation to anatomically adjacent pelvic nodal regions, producing a reproducible regional hierarchy of nodal dose that is largely independent of IMRT delivery technique. While VMAT improves treatment efficiency, the consistency of regional dose patterns across techniques suggests that incidental nodal exposure may be influenced more by target geometry than by the delivery technique. These findings provide a framework for future studies evaluating the clinical significance of incidental pelvic nodal irradiation.

## CRediT authorship contribution statement

**Birhan Demirhan:** Writing – review & editing, Supervision, Methodology, Investigation, Formal analysis, Conceptualization. **Aysenur Elmali:** Writing – review & editing, Visualization, Investigation, Data curation. **Recep Bozca:** Writing – review & editing, Validation, Investigation, Data curation. **Yemliha Dolek:** Writing – review & editing, Investigation, Formal analysis. **Ozan Cem Guler:** Writing – original draft, Visualization, Supervision, Methodology, Data curation, Conceptualization. **Cem Onal:** Writing – review & editing, Supervision, Project administration, Data curation, Conceptualization.

## Ethics declaration

### Informed consent and patient details

Written informed consent to take part in the study and to publish the article has been obtained from all participants or their legal representatives. The privacy rights of participants have been observed.

### Studies in human

This study was performed in compliance with relevant laws, regulatory frameworks and guidelines where the research took place.

Ethics committee approval was not required under relevant laws and institutional guidelines. Ethical approval and informed consent were not required because this retrospective dosimetric study used fully anonymized planning CT datasets and involved no additional intervention or collection of identifiable patient information, in accordance with applicable regulations and institutional guidelines.

## Declaration of competing interest

The authors declare that they have no known competing financial interests or personal relationships that could have appeared to influence the work reported in this paper.
